# Neuroinflammatory Cytokines Induce Amyloid Beta Neurotoxicity through Modulating Amyloid Precursor Protein Levels/Metabolism

**DOI:** 10.1155/2018/3087475

**Published:** 2018-10-25

**Authors:** Fawaz Alasmari, Musaad A. Alshammari, Abdullah F. Alasmari, Wael A. Alanazi, Khalid Alhazzani

**Affiliations:** Department of Pharmacology and Toxicology, College of Pharmacy, King Saud University, Riyadh, Saudi Arabia

## Abstract

Neuroinflammation has been observed in association with neurodegenerative diseases including Alzheimer's disease (AD). In particular, a positive correlation has been documented between neuroinflammatory cytokine release and the progression of the AD, which suggests these cytokines are involved in AD pathophysiology. A histological hallmark of the AD is the presence of beta-amyloid (A*β*) plaques and tau neurofibrillary tangles. Beta-amyloid is generated by the sequential cleavage of beta (*β*) and gamma (*γ*) sites in the amyloid precursor protein (APP) by *β*- and *γ*-secretase enzymes and its accumulation can result from either a decreased A*β* clearance or increased metabolism of APP. Previous studies reported that neuroinflammatory cytokines reduce the efflux transport of A*β*, leading to elevated A*β* concentrations in the brain. However, less is known about the effects of neuroinflammatory mediators on APP expression and metabolism. In this article, we review the modulatory role of neuroinflammatory cytokines on APP expression and metabolism, including their effects on *β*- and *γ*-secretase enzymes.

## 1. Introduction

The progression of neurodegenerative disorders including Alzheimer's disease (AD) leads to death and other negative health consequences [1–3]. The prevalence of AD increases significantly with increasing age in the United States (US) [4]. Importantly, it has been estimated that, by 2050, the population of AD patients in the US will increase threefold over the number in 2000 [5]. Moreover, studies have found that more than 4.5 million new cases of dementia arise per year, 70% of which are attributed to AD [6, 7]. In the US, AD costs over $170 billion every year [8]; reducing the progression of AD could provide beneficial consequences clinically and economically. Finding molecular therapeutic targets involved in the progression of AD is therefore highly important and studies exploring its causative factors are needed.

AD is accompanied by the formation of beta-amyloid (A*β*) plaques, neurodegeneration, and neuroinflammation [for review, see [11]]. Immune cells may produce autoimmune inflammation, which leads to the progression of neurodegenerative diseases including AD [for review, see [12]]. Triggering receptors expressed on myeloid cells 2 in microglial receptors for A*β* have been found to regulate the physiological and pathological functions of microglia [13]. A significant increase in neuroinflammatory cytokines release has been observed in animal models of AD [14]; these cytokines can cause neurodegeneration [15] and activation of microglia [16] that might further progress AD. The accumulation of A*β* in the brain also increases production of neuroinflammatory cytokines [14, 17]. Studies suggest that attenuation of AD symptoms is attributable at least in part to restored concentrations of neuroinflammatory cytokines, indicating that these molecules have a critical role in disease development [for review, see [18]]. Furthermore, the exposure of human neuronal and extraneuronal cells to an inflammatory cytokine such as interleukin-18 (IL-18) or a combination of interferon-*γ* (IFN-*γ*) and tumor necrosis factor-*α* (TNF-*α*) has been shown to lead to increased A*β* production [19, 20]. This indicates that these cytokines modulate proteins that are responsible for generating A*β* [20]. The exposure to inflammatory cytokines also reduces A*β* transport [21, 22], which might lead to accumulation of A*β* in the brain. This was confirmed by a later study, which showed that an anti-inflammatory agent reduced the accumulation of A*β* through upregulating ATP-binding cassette-B1 (ABCB1) [23], a protein involved in the clearance of A*β* from the brain into the vascular system [24, 25]. A recent opinion article discussed the role of ABCB1 in AD progression through modulation of A*β* uptake [26].

**Figure 1:**
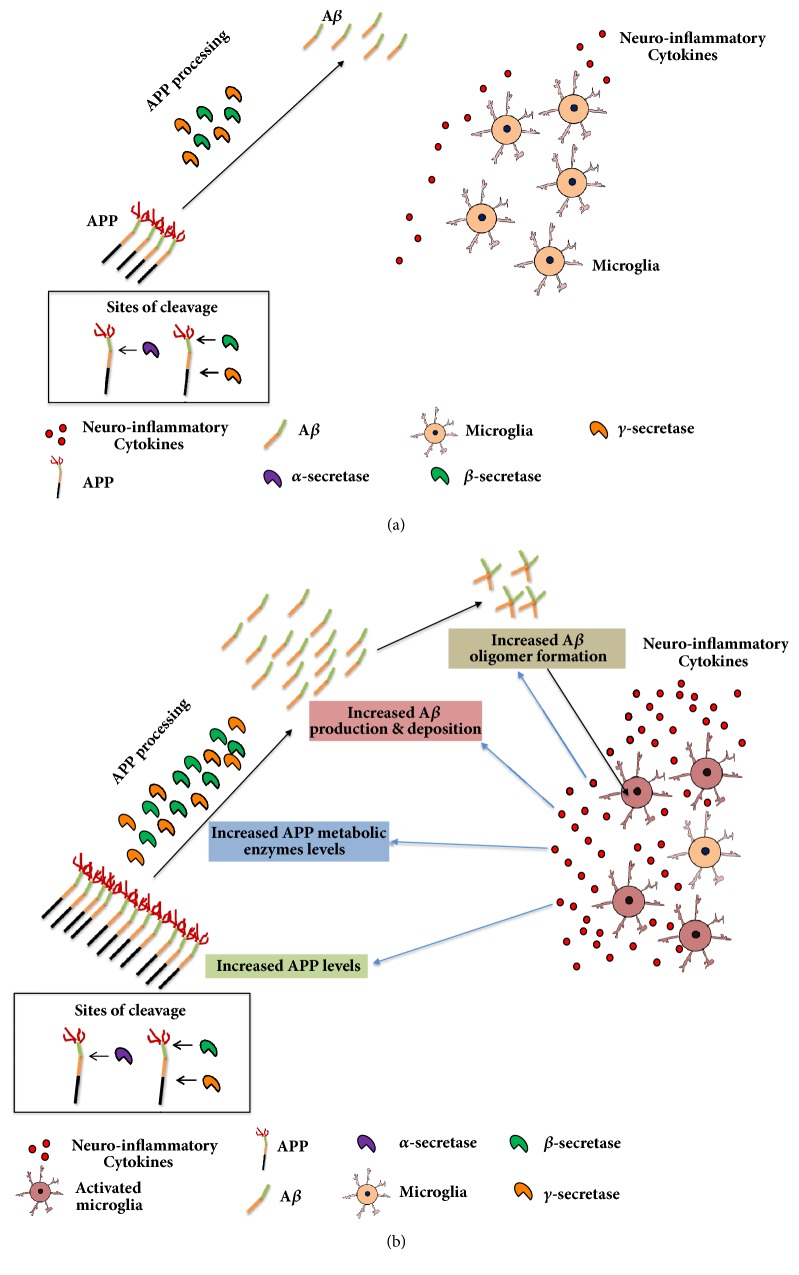
Schematic diagram for the effects of neuroinflammatory cytokines on amyloid precursor protein (APP) processing and beta-amyloid (A*β*) production. Amyloid precursor proteins (APP) are cleaved by beta (*β*) and gamma (*γ*) sites in the APP by *β*- and *γ*-secretase enzymes producing A*β* in the brain. (a) Normal levels and activity of APP, APP metabolic enzymes, and neuroinflammatory cytokines in control brain. (b) In Alzheimer disease (AD), A*β* is accumulated in the brain leading to formation of A*β* oligomers. This effect leads to activation of microglia, which increases the production of neuroinflammatory cytokines. These cytokines increase APP levels, upregulate *β*-secretase and *γ*-secretase, and decrease A*β* clearance in the brain. These effects result in further increase in A*β* concentrations and formation of A*β* oligomers and plaques.

A*β* accumulation in the brain is one of the histological hallmarks associated with AD [for review, see [27]]. A*β* is formed through the sequential cleavage of the amyloid precursor protein (APP) by beta (*β*-) and gamma (*γ*-) secretase enzymes [28, 29]. Prior exposure to neuroinflammatory cytokines has been associated with significant increases in the expression of APP in neuronal and glial cells [30] (Figure 1(b)). The regulatory role of neuroinflammatory cytokines on secretase enzymes has been discussed in previous reviews [31, 32]. In this article, we highlighted the effects of neuroinflammatory cytokines on APP processing including APP secretases as well as A*β* production and accumulation.

Regarding the effects of neuroinflammation on APP processing in human brain samples, studies have found that APP levels and metabolism are altered in postmortem brain tissues from AD patients [33, 34]. A study reported that APP mRNA and protein expression level are increased in postmortem human temporal neocortex of AD patients [34]. Additionally, the activity and protein expression of *β*-secretase are increased in the neocortex of AD patients [33], suggesting that the formation of A*β* is further increased, leading to pathogenesis. For example, IL-1 levels are increased in the postmortem samples of hippocampus, thalamus, hypothalamus, and cortex of AD patients compared to those obtained from both individuals with vascular dementia and controls [35]. However, the effects of neuroinflammatory cytokines on APP cleaving enzymes merit further investigation. Since neuroinflammation is a common symptom associated with AD, we discuss herein the modulatory role of neuroinflammatory cytokines on APP expression and metabolism in AD models.

### 1.1. APP

APP is a protein expressed ubiquitously in human body and APP brain isoform is processed into A*β* and mainly localized in the neurons and synapses [36]. APP is the precursor of soluble APP-*α* and soluble APP-*β* through cleavage by *α*-secretase and *β*-secretase, respectively. A*β* is generated from the APP through sequential cleavage at the *β* and *γ* sites of the APP via *β*- and *γ*-secretase enzymes, respectively [28, 29]. Physiologically, it is involved in modulating neuronal development including differentiation, migration, and synaptogenesis, suggesting that APP is essential for maintaining homeostasis and synaptic plasticity [37–40]. In this article, we focus on the role of APP and its therapeutic implications in the AD.

#### 1.1.1. Role of APP Expression/Metabolism in AD

The expression of APP cleaving enzymes is increased in the brain cortex of AD patients [41] and the mRNA expression of APP was also increased in AD rat models [42], and these effects are also associated with elevated brain A*β* concentrations [for review, see [43]]. It has been suggested that the activities of *β*- and *γ*-secretase enzymes are enhanced in the brains of AD individuals [33, 44], which could elevate brain A*β* levels leading to formation of senile plaques (A*β* deposit) [for review, see [45]]. Pharmacological targeting of *β*- and *γ*-secretase could, therefore, attenuate APP-increased A*β* concentrations [46, 47], which might reduce AD-associated symptoms in AD animal models. This hypothesis is supported by reviews indicating that *β*- and *γ*-secretase inhibitors attenuate the behavioral symptoms of AD in animals [48, 49]. Specifically, the expression and functions of these enzymes were altered in the brains of AD treated model [33, 41, 50]. Importantly, a *γ*-secretase enzyme modulator, CHF5074, has been found to attenuate the number and occupation area of A*β* plaques in the cortex and hippocampus of an AD model, and improved AD-associated behavioral symptoms were demonstrated using swimming path test [51]. This study found that CHF5074 also reduced plaques-occupied area in microglia suggesting that this compound can attenuate neuroinflammation associated with AD. However, studies are warranted to explore the effects of neuroinflammatory cytokines on the activity and expression of *β*- and *γ*-secretase enzymes in AD models.

In addition to the role of APP metabolism in AD, the overexpression of APP has been used extensively to develop AD models [52, 53]. A previous study found that APP expression level was increased in AD models [34, 54] indicating that increase in APP expression may cause accumulation of A*β* in the brain. Interestingly, exposure to neuroinflammatory cytokines increased the expression of APP using neuronal and nonneuronal cell lines [20, 30, 55]. These findings suggest that neuroinflammatory cytokines could cause accumulation of A*β* in the brain either through increased expression of APP or by decreasing the transport of A*β* into the vascular system. It is important to note that both protein and mRNA expression of APP were upregulated [34], and other reports have also shown that apolipoprotein E4 (APOE4) secreted glia stimulated APP transcription and A*β* production in human cultured neurons [56]. This suggests that these inflammatory cytokines might upregulate APP through modulating the expression of its transcription factors; however, this hypothesis needs further investigation. In this article, we review the effects of proinflammatory cytokines produced in AD models on the mRNA and protein expression of APP.

#### 1.1.2. APP Processing as a Therapeutic Target for Attenuating AD Pathology

As discussed earlier, APP metabolism is a process involved in the production of A*β* in the brain [28, 29]. Therefore, modulating enzymes that cleave APP into A*β* could attenuate the concentrations of A*β* [51, 57–59], which might lead to the attenuation of AD behavioral symptoms [51, 58]. The daily treatment of transgenic model of the AD with a *γ*-secretase enzyme modulator for seven months has been shown to reduce the concentrations of A*β* in the cortex and hippocampus [57]. In another study, mice with AD that were exposed to methylene blue for three months also had decreased productions and concentrations of A*β*1–40 and A*β*1–42 in the cortex via inhibitory effects on the *β*-secretase enzyme [58]. This effect was associated with the attenuation of AD behavioral symptoms such as cognition, locomotion, and rearing. Similarly, a three-month treatment of transgenic AD mice model with selenomethionine decreased the deposition and production of A*β* in the cortex and hippocampus through modulating *β*-secretase enzyme in the same brain regions [59]. Together, these findings indicate that modulating either secretase enzymes attenuates A*β* plaques and consequently the associated behavioral symptoms.

Targeting the expression of APP is another therapeutic strategy for attenuating A*β* deposition [60, 61] and therefore potentially limiting AD progression. In CHO APP_751SW_ cells treated with 2-[(pyridine-2-ylmethyl)-amino]-phenol (2-PMAP), both APP level and the production of A*β*x-_40_ and A*β*x-_42_ were inhibited in a dose-dependent manner [60]. This study also found that the inhibitory effect of 2-PMAP on APP was realized through a mechanism affecting APP translation. In addition, 2-PMAP was able to reduce the length of APP and its fragmentation [60]. These effects were associated with improved memory performance in a transgenic mouse model. Another study found that treatment of an AD mouse model with icariin, a compound from Chinese herb* Epimedium* spp., reduced the burden and production of A*β* in the hippocampus through reducing the levels of APP and *β*-secretase [61]. These studies confirmed that APP level plays a crucial role in the deposition of A*β* in the brain and therefore in neurodegeneration and neuroinflammation. A novel approach would focus on targeting APP modifications. The selective inhibition of APP colocalization with BACE1 and *γ*-secretase could lead to a couple of therapeutic benefits. First, it would have minimal impact on substrates acting by binding to *β*- and *γ*-secretase. Second, it would directly affect APP trafficking leading to reducing in the colocalization of APP with *β*- and *γ*-secretase [62, 63]. Thus, modulating the levels of APP and secretase enzymes are promising therapeutic targets for attenuating AD pathogenesis.

### 1.2. Role of Neuroinflammatory Cytokines in AD

Increased production of neuroinflammatory cytokines is broadly associated with neurological disorders, including AD [20, 64, 65]. For instance, high concentrations of IL-1, IL-17, IFN-*γ*, and TNF-*α* are observed in the brains of AD models [35, 66, 67]. Moreover, increased secretion of IL-17 and IFN-*γ* from T cells has been demonstrated in a transgenic AD mouse model, and treatment with anti-IFN-*γ* antibody has been shown to attenuate A*β* deposition induced by CD4(+) T cells [66]. In addition, increased neuroinflammatory cytokines levels have been observed in human brain tissues [35]. Moreover, the combination of adeno-associated virus 1 expressing murine IL-6 and an A*β* suppressor induced further improvement in plaque clearance in transgenic mice [68]. Additionally, decoy receptor 3 (DcR3), a TNF soluble protein, has been found to reduce the accumulation of amyloid plaque, an effect associated with reduced A*β*-increased anti-inflammatory cytokines production from microglia [69]. It is important to note that overexpression of IL-17A, a marker around deposits of accumulated A*β*, reduced the level of A*β* in the hippocampus and cerebrospinal fluid. This effect was associated with increased ABCA1 expression; a protein transports A*β* from brain into the circulatory system [70]. High concentrations of TNF-*α* were observed in the hippocampus of a mouse AD model, which induced synaptic plasticity and transmission of glutamate [67]. These data provide evidence for targets of neuroinflammatory cytokines that may also provide pharmacological targets for attenuating the pathogenesis of AD.

To support these findings, targeting neuroinflammatory cytokines has been shown to attenuate AD behavioral symptoms in animals [66, 67, 71, 72]. A study found that anti-IFN-*γ* attenuated impaired cognition in a transgenic AD mouse model [66]. Another model study showed that pretreatment with a TNF-*α* inhibitor prevented synaptic deficiency induced by increased secretion of TNF-*α* in the hippocampus [67]. Moreover, treatment with a TNF-*α* synthesis inhibitor, 3,6′-dithiothalidomide, attenuated impaired memory associated with neuroinflammation and also reduced neurogenesis in the hippocampus that had been induced by A*β* peptide [71]. Pretreatment with 3,6′-dithiothalidomide of an AD mouse model also attenuated A*β* peptide-induced memory deficiency, while chronic exposure reduced impaired cognition and memory dysfunction, effects that were so associated with reduced APP and A*β* plaque levels [73]. Furthermore, attenuation of memory dysfunction has been observed in mice treated with a TNF-*α* receptor inhibitor [72]. Taken together, these results suggest that neuroinflammatory cytokines in general and TNF-*α*, in particular, are implicated in AD pathogenesis.

## 2. Effects of Neuroinflammatory Cytokines on APP in AD Models

From our previous section, there is evidence that exposure to neuroinflammatory cytokines may modulate APP levels [73, 74] and metabolism [75, 76] (Figure 1(b)) which might be targeted for attenuating AD progression. These effects were not observed with the control group (Figure 1(a)). For example, increased APP expression has been found to lead to the increase of brain A*β* levels [60]. In addition, modulation of APP metabolism attenuated deposition of A*β* [57], which might lead to improved AD-behavioral symptoms.

### 2.1. Effects of Neuroinflammatory Cytokines on APP Expression/Level

Several studies found that APP expression is a key factor involved in the development of AD [34, 52]. Importantly, the expression/level of APP is positively correlated with the concentrations of A*β* in the brain [60, 61]. Studies have been performed to determine the causative factors involved in increased APP expression. In AD model, the neuroinflammatory cytokines have been found to modulate the expression level of APP [30]. This suggests that neuroinflammation associated with AD plays a substantial role in elevating the expression level of the APP which leads to increased A*β* production. This effect results in the formation of A*β* plaque and oligomers that lead to neurodegeneration, an effect involved in AD pathogenesis [for review, see [77]].

Supporting the hypothesis that neuroinflammation modulates AD development, the exposure to neuroinflammatory cytokines increases APP expression [30], which might modulate the level of A*β* in animals developing AD. A prior study reported that the exposure to IL-1*β* and IL-1 increased the mRNA expression of APP in endothelial and neuronal cells [30]. Similarly, the exposure to IL-1 increased the level of APP transcripts in endothelial cells from human umbilical veins [78]. We suggest here that the synthesis of APP is modulated by neuroinflammatory cytokines including IL-1, TNF-*α*, and IFN-*γ* [78–80]. A study found that the level of APP constitutive shedding is regulated by TNF-*α* converting enzyme [79]. Moreover, the exposure to TNF-*α* or IFN-*γ* upregulated APP in both neurons and astrocytes [80]. These findings provide evidence for the potential effects of neuroinflammation on the amyloid precursor system in the progression of the AD.

### 2.2. Neuroinflammatory Cytokines Modulate APP Metabolism

Neuroinflammatory cytokines have also been shown to modulate APP metabolic enzymes including *β*- and *γ*-secretase [75, 76, 80–82]. Interleukins, TNF-*α*, and IFN-*γ* are known to stimulate *γ*-secretase enzyme activity; this effect was associated with increased production of A*β* and the APP intracellular domain [75]. TNF-*α* and IFN-*γ* have been shown to enhance *β*-secretase enzyme expression in a transgenic mouse model of the AD, increasing A*β* deposition and reducing its uptake [76]. Treatment with sulindac sulfide, an anti-inflammatory agent, restored lipopolysaccharide (LPS)-induced *β*-secretase expression in neuron cells [80]. In addition, this study found that this compound could reduce the secretion of A*β*_42_ in neurons treated with LPS_._ Furthermore, genetic deletion of the TNF-*α* death receptor was associated with reduced *β*-secretase enzyme activity and expression in AD mice [81]. This effect was also associated with reduced microglia activation and reduced A*β* production and deposits. Further support comes from 5XFAD/TNF-*α*^−/−^ mice, which have significantly decreased protein expression of *β*-secretase enzyme and A*β* deposition [82]. Finally, the TNF-*α* converting enzyme has been found to regulate *γ*-secretase enzyme activity [83]. These data shed light on the potential effects of inflammatory cytokines on *β*- and *γ*-secretase and A*β* system dysregulation in AD models. More studies are required to investigate whether these neuroinflammatory cytokines could be pharmacologically targeted to attenuate AD-associated symptoms.

## 3. Conclusion

Neuroinflammation associated with AD is suggested to progress AD in part by increasing the accumulation of A*β* in the brain, which induces A*β* plaques and leads to neurodegeneration and microglial activation. Moreover, the release of neuroinflammatory cytokines is increased in AD models; these cytokines reduce the clearance of A*β* and increase its production, in part by increasing the expression/levels of APP (Figure 1(b)). The cytokines also modulate APP metabolism leading to AD pathogenesis. Studies are warranted to investigate the effects of compounds that have anti-inflammatory properties on AD progression.
